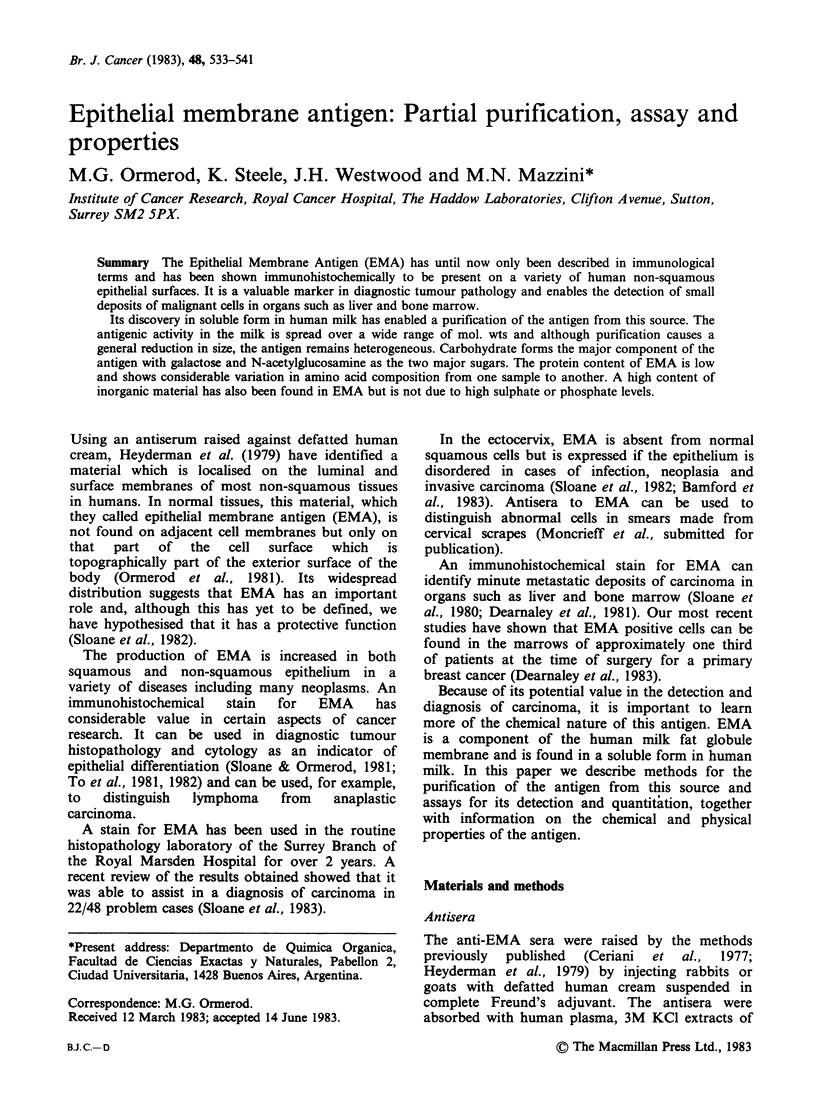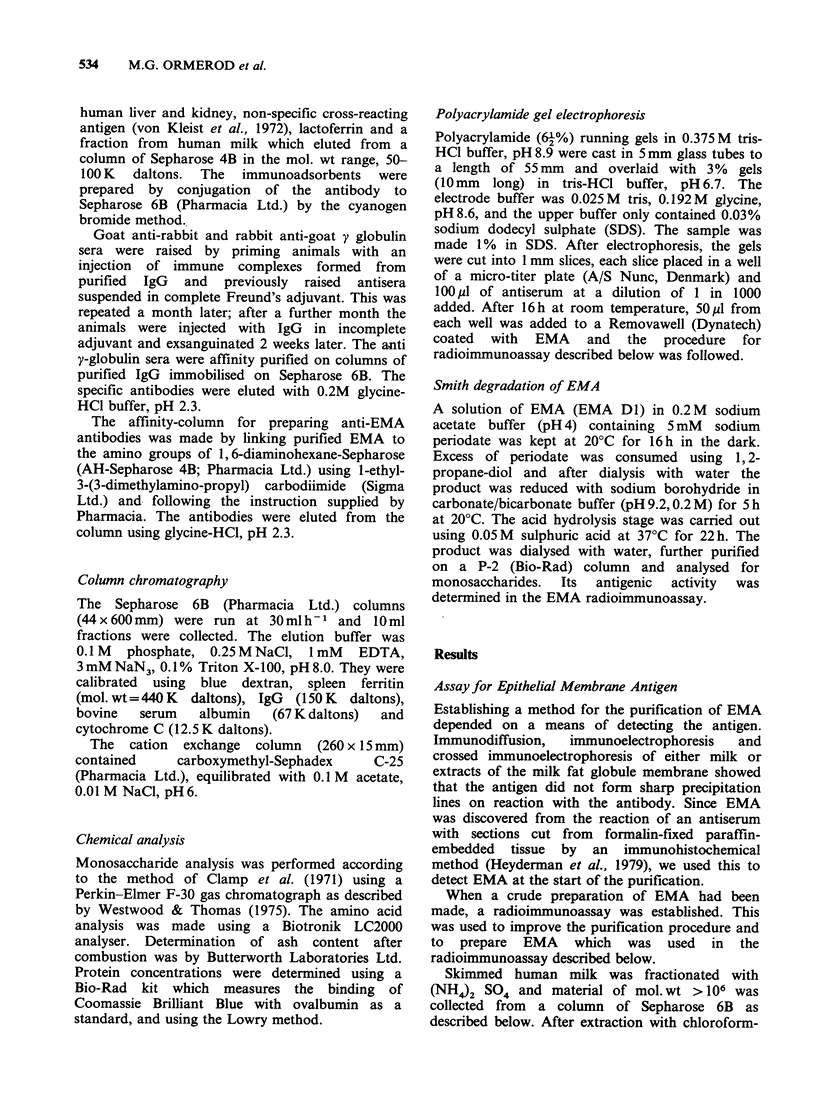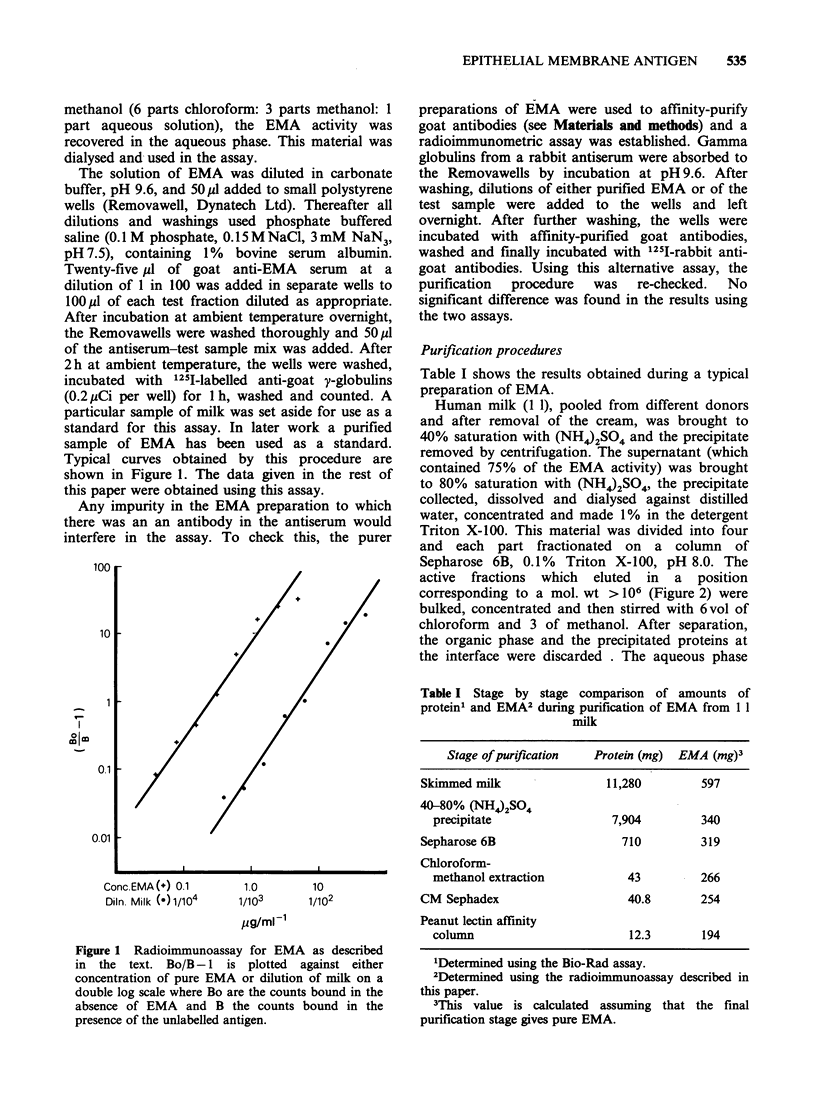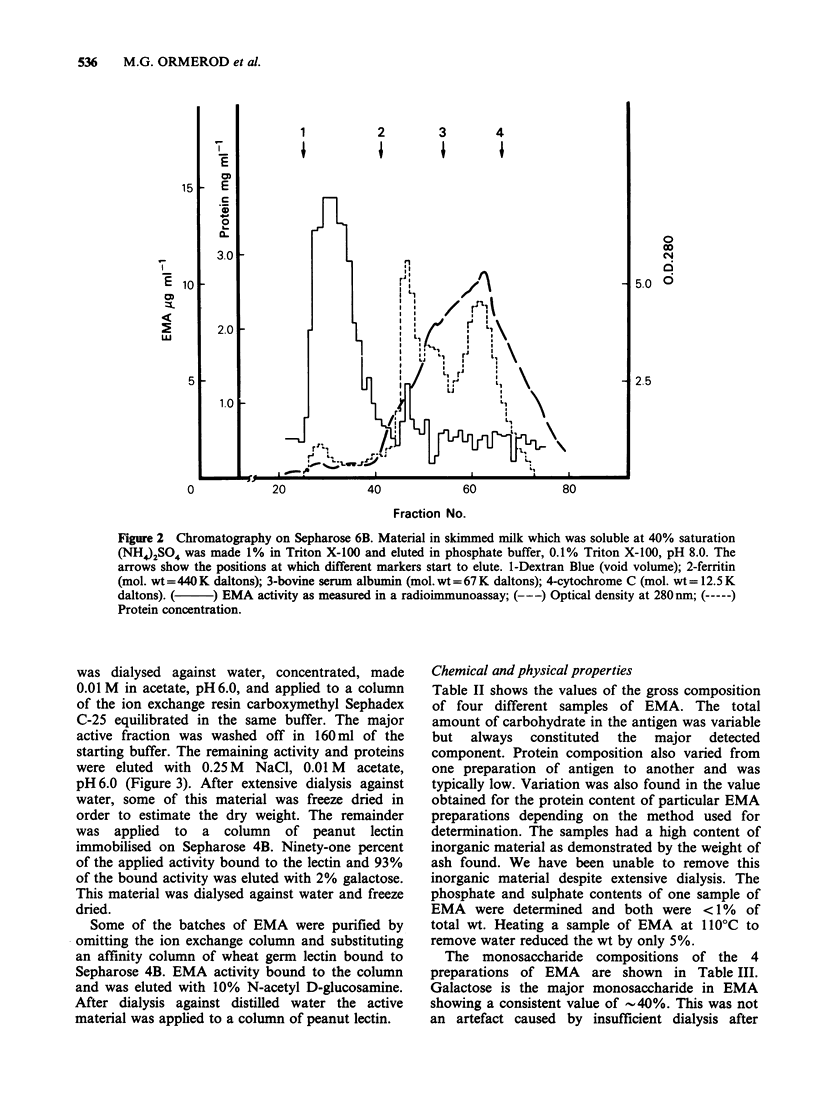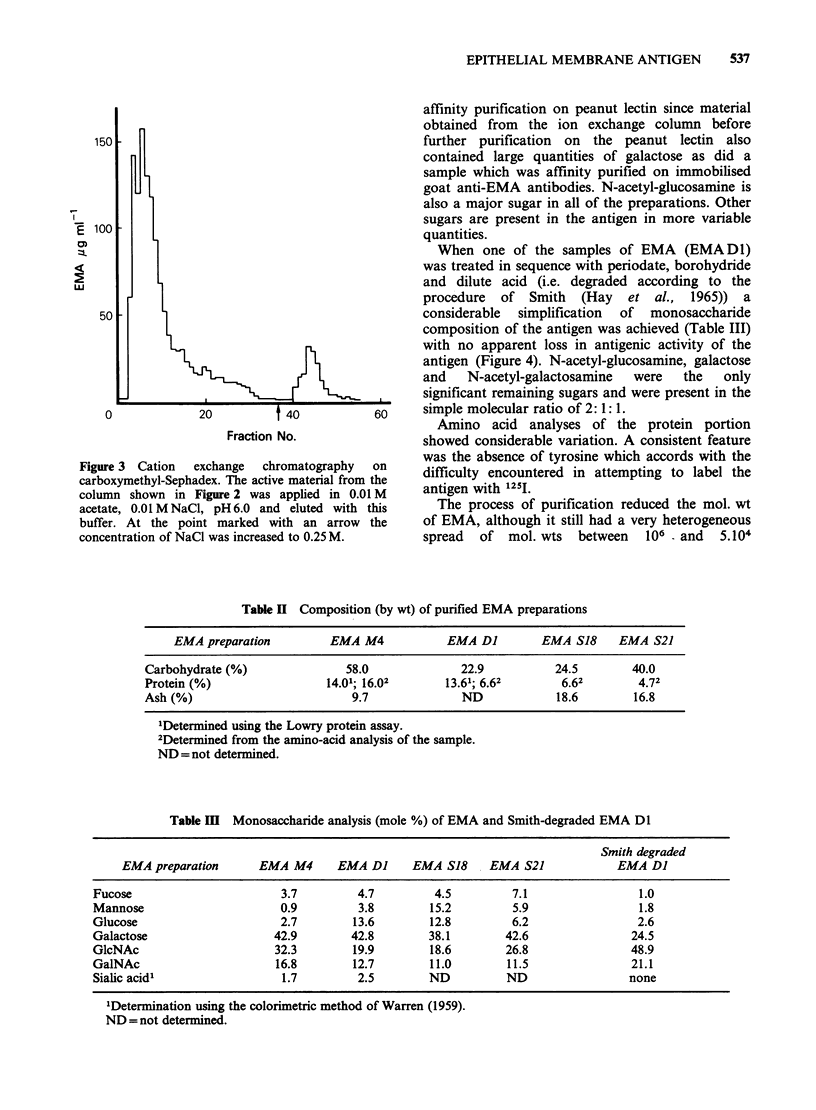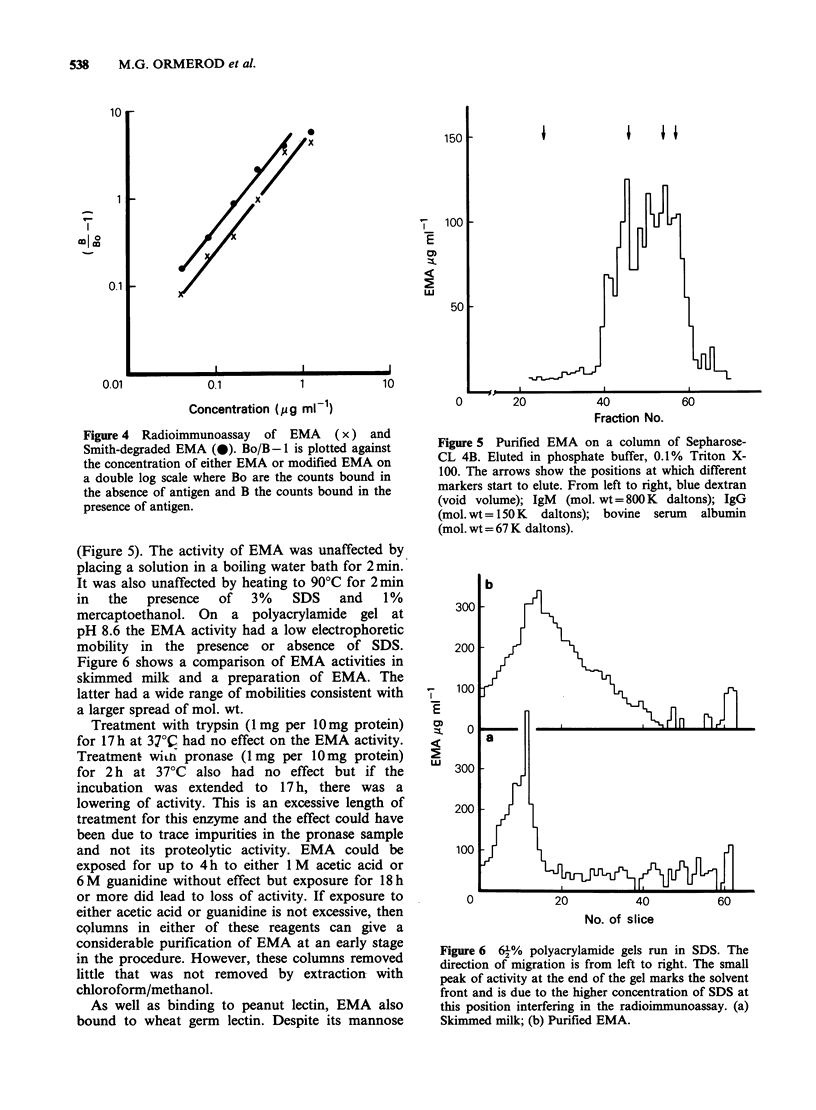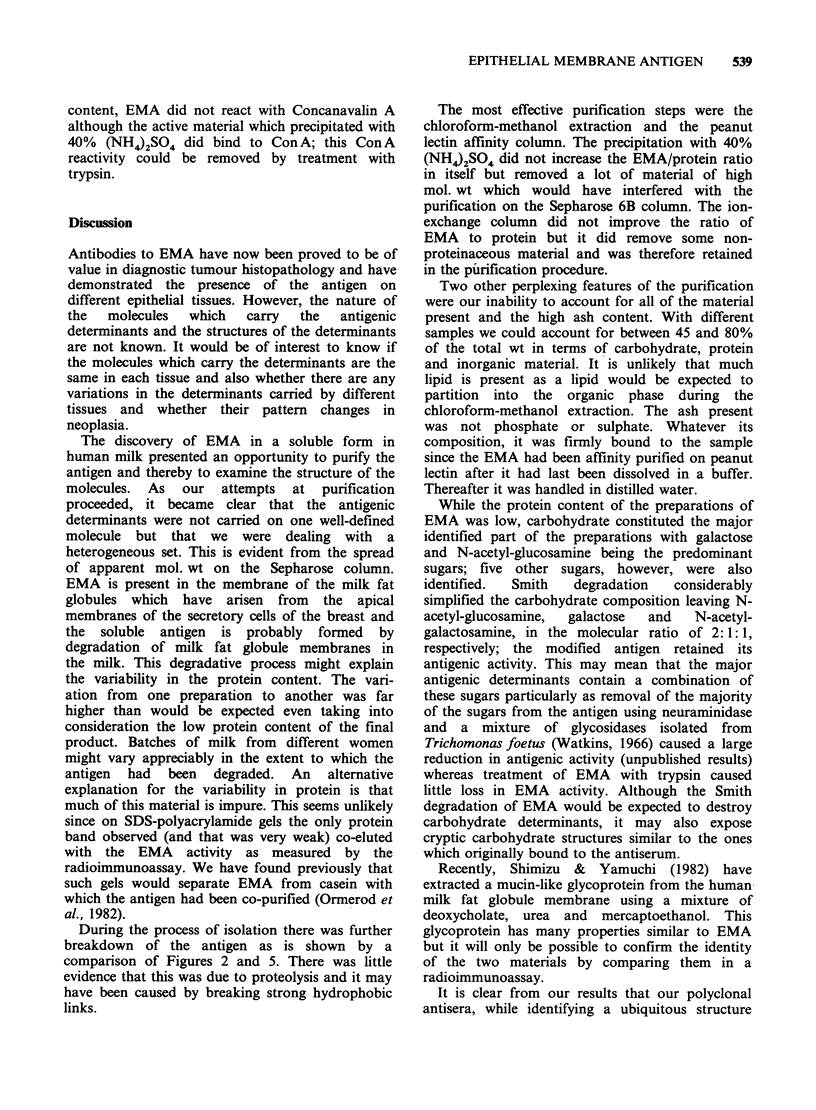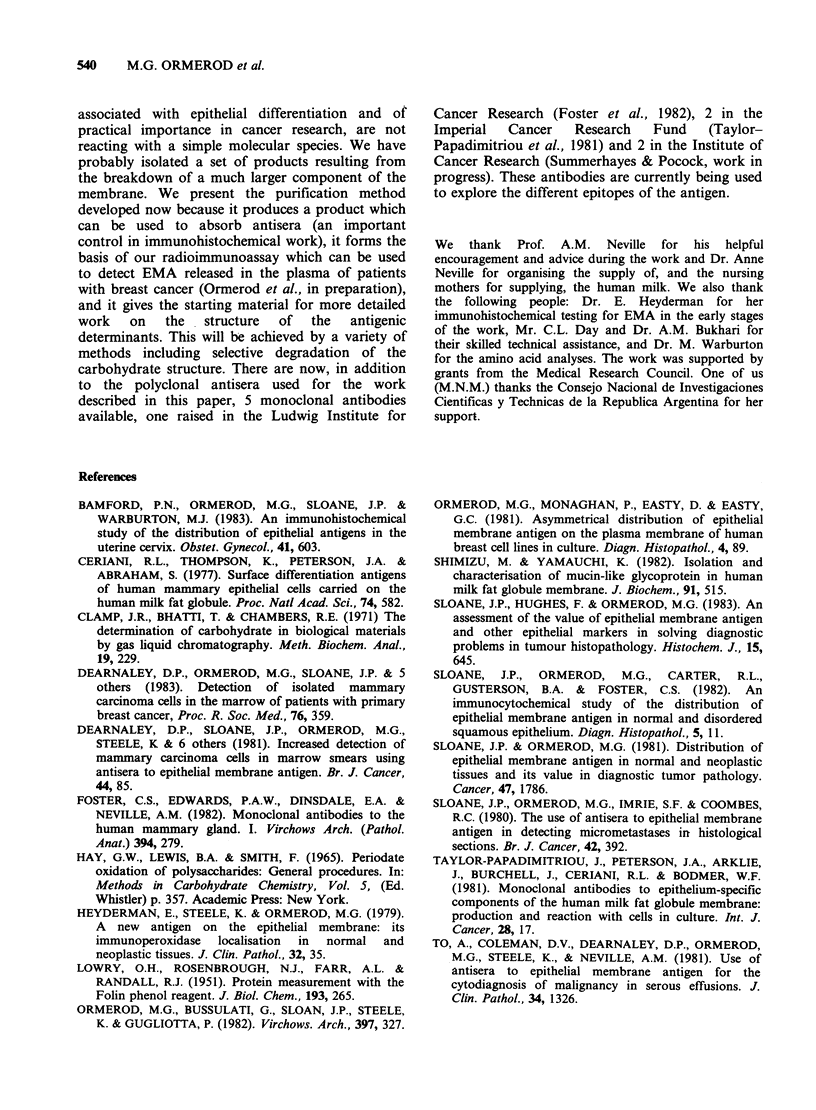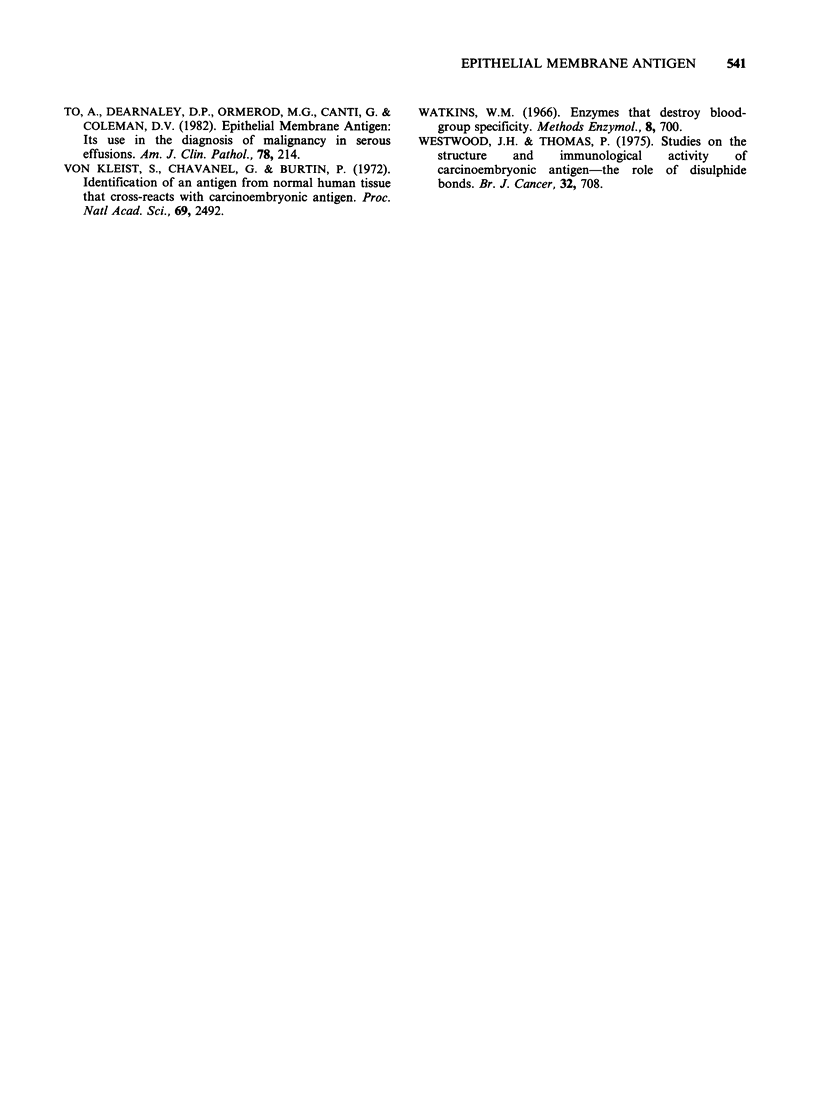# Epithelial membrane antigen: partial purification, assay and properties.

**DOI:** 10.1038/bjc.1983.226

**Published:** 1983-10

**Authors:** M. G. Ormerod, K. Steele, J. H. Westwood, M. N. Mazzini

## Abstract

The Epithelial Membrane Antigen (EMA) has until now only been described in immunological terms and has been shown immunohistochemically to be present on a variety of human non-squamous epithelial surfaces. It is a valuable marker in diagnostic tumour pathology and enables the detection of small deposits of malignant cells in organs such as liver and bone marrow. Its discovery in soluble form in human milk has enabled a purification of the antigen from this source. The antigenic activity in the milk is spread over a wide range of mol. wts and although purification causes a general reduction in size, the antigen remains heterogeneous. Carbohydrate forms the major component of the antigen with galactose and N-acetylglucosamine as the two major sugars. The protein content of EMA is low and shows considerable variation in amino acid composition from one sample to another. A high content of inorganic material has also been found in EMA but is not due to high sulphate or phosphate levels.


*Br. J. Cancer* (1983), **48**, 533–541

### Epithelial membrane antigen: Partial purification, assay and properties

M.G. Ormerod, K. Steele, J.H. Westwood and M.N. Mazzini*

*Institute of Cancer Research, Royal Cancer Hospital, The Haddow Laboratories, Clifton Avenue, Sutton, Surrey SM2 5PX.*

**Summary** The Epithelial Membrane Antigen (EMA) has until now only been described in immunological terms and has been shown immunohistochemically to be present on a variety of human non-squamous epithelial surfaces. It is a valuable marker in diagnostic tumour pathology and enables the detection of small deposits of malignant cells in organs such as liver and bone marrow.

Its discovery in soluble form in human milk has enabled a purification of the antigen from this source. The antigenic activity in the milk is spread over a wide range of mol. wts and although purification causes a general reduction in size, the antigen remains heterogeneous. Carbohydrate forms the major component of the antigen with galactose and N-acetylglucosamine as the two major sugars. The protein content of EMA is low and shows considerable variation in amino acid composition from one sample to another. A high content of inorganic material has also been found in EMA but is not due to high sulphate or phosphate levels.

Using an antiserum raised against defatted human cream, Heyderman *et al.* (1979) have identified a material which is localised on the luminal and surface membranes of most non-squamous tissues in humans. In normal tissues, this material, which they called epithelial membrane antigen (EMA), is not found on adjacent cell membranes but only on that part of the cell surface which is topographically part of the exterior surface of the body (Ormerod *et al.*, 1981). Its widespread distribution suggests that EMA has an important role and, although this has yet to be defined, we have hypothesised that it has a protective function (Sloane *et al.*, 1982).

The production of EMA is increased in both squamous and non-squamous epithelium in a variety of diseases including many neoplasms. An immunohistochemical stain for EMA has considerable value in certain aspects of cancer research. It can be used in diagnostic tumour histopathology and cytology as an indicator of epithelial differentiation (Sloane & Ormerod, 1981; To *et al.*, 1981, 1982) and can be used, for example, to distinguish lymphoma from anaplastic carcinoma.

A stain for EMA has been used in the routine histopathology laboratory of the Surrey Branch of the Royal Marsden Hospital for over 2 years. A recent review of the results obtained showed that it was able to assist in a diagnosis of carcinoma in 22/48 problem cases (Sloane *et al.*, 1983).

*Present address: Departmento de Quimica Organica, Facultad de Ciencias Exactas y Naturales, Pabellon 2, Ciudad Universitaria, 1428 Buenos Aires, Argentina.

Correspondence: M.G. Ormerod.

Received 12 March 1983; accepted 14 June 1983.

In the ectocervix, EMA is absent from normal squamous cells but is expressed if the epithelium is disordered in cases of infection, neoplasia and invasive carcinoma (Sloane *et al.*, 1982; Bamford *et al.*, 1983). Antisera to EMA can be used to distinguish abnormal cells in smears made from cervical scrapes (Moncrieff *et al.*, submitted for publication).

An immunohistochemical stain for EMA can identify minute metastatic deposits of carcinoma in organs such as liver and bone marrow (Sloane *et al.*, 1980; Dearnaley *et al.*, 1981). Our most recent studies have shown that EMA positive cells can be found in the marrows of approximately one third of patients at the time of surgery for a primary breast cancer (Dearnaley *et al.*, 1983).

Because of its potential value in the detection and diagnosis of carcinoma, it is important to learn more of the chemical nature of this antigen. EMA is a component of the human milk fat globule membrane and is found in a soluble form in human milk. In this paper we describe methods for the purification of the antigen from this source and assays for its detection and quantitation, together with information on the chemical and physical properties of the antigen.

### Materials and methods

### *Antisera*

The anti-EMA sera were raised by the methods previously published (Ceriani *et al.*, 1977; Heyderman *et al.*, 1979) by injecting rabbits or goats with defatted human cream suspended in complete Freund's adjuvant. The antisera were absorbed with human plasma, 3M KCl extracts of

© The Macmillan Press Ltd., 1983

human liver and kidney, non-specific cross-reacting antigen (von Kleist et al., 1972), lactoferrin and a fraction from human milk which eluted from a column of Sepharose 4B in the mol. wt range, 50–100 K daltons. The immunoadsorbents were prepared by conjugation of the antibody to Sepharose 6B (Pharmacia Ltd.) by the cyanogen bromide method.

Goat anti-rabbit and rabbit anti-goat $\gamma$ globulin sera were raised by priming animals with an injection of immune complexes formed from purified IgG and previously raised antisera suspended in complete Freund's adjuvant. This was repeated a month later; after a further month the animals were injected with IgG in incomplete adjuvant and exsanguinated 2 weeks later. The anti $\gamma$-globulin sera were affinity purified on columns of purified IgG immobilised on Sepharose 6B. The specific antibodies were eluted with 0.2M glycine-HCl buffer, pH 2.3.

The affinity-column for preparing anti-EMA antibodies was made by linking purified EMA to the amino groups of 1,6-diaminohexane-Sepharose (AH-Sepharose 4B; Pharmacia Ltd.) using 1-ethyl-3-(3-dimethylamino-propyl) carbodiimide (Sigma Ltd.) and following the instruction supplied by Pharmacia. The antibodies were eluted from the column using glycine-HCl, pH 2.3.

*Column chromatography*

The Sepharose 6B (Pharmacia Ltd.) columns (44 x 600 mm) were run at 30 ml h$^{-1}$ and 10 ml fractions were collected. The elution buffer was 0.1 M phosphate, 0.25 M NaCl, 1 mM EDTA, 3 mM $NaN_3$, 0.1% Triton X-100, pH 8.0. They were calibrated using blue dextran, spleen ferritin (mol. wt = 440 K daltons), IgG (150 K daltons), bovine serum albumin (67 K daltons) and cytochrome C (12.5 K daltons).

The cation exchange column (260 x 15 mm) contained carboxymethyl-Sephadex C-25 (Pharmacia Ltd.), equilibrated with 0.1 M acetate, 0.01 M NaCl, pH 6.

*Chemical analysis*

Monosaccharide analysis was performed according to the method of Clamp et al. (1971) using a Perkin-Elmer F-30 gas chromatograph as described by Westwood & Thomas (1975). The amino acid analysis was made using a Biotronik LC2000 analyser. Determination of ash content after combustion was by Butterworth Laboratories Ltd. Protein concentrations were determined using a Bio-Rad kit which measures the binding of Coomassie Brilliant Blue with ovalbumin as a standard, and using the Lowry method.

*Polyacrylamide gel electrophoresis*

Polyacrylamide ($6\frac{1}{2}$%) running gels in 0.375 M tris-HCl buffer, pH 8.9 were cast in 5 mm glass tubes to a length of 55 mm and overlaid with 3% gels (10 mm long) in tris-HCl buffer, pH 6.7. The electrode buffer was 0.025 M tris, 0.192 M glycine, pH 8.6, and the upper buffer only contained 0.03% sodium dodecyl sulphate (SDS). The sample was made 1% in SDS. After electrophoresis, the gels were cut into 1 mm slices, each slice placed in a well of a micro-titer plate (A/S Nunc, Denmark) and 100 $\mu$l of antiserum at a dilution of 1 in 1000 added. After 16 h at room temperature, 50 $\mu$l from each well was added to a Removawell (Dynatech) coated with EMA and the procedure for radioimmunoassay described below was followed.

*Smith degradation of EMA*

A solution of EMA (EMA D1) in 0.2 M sodium acetate buffer (pH 4) containing 5 mM sodium periodate was kept at 20°C for 16 h in the dark. Excess of periodate was consumed using 1,2-propane-diol and after dialysis with water the product was reduced with sodium borohydride in carbonate/bicarbonate buffer (pH 9.2, 0.2 M) for 5 h at 20°C. The acid hydrolysis stage was carried out using 0.05 M sulphuric acid at 37°C for 22 h. The product was dialysed with water, further purified on a P-2 (Bio-Rad) column and analysed for monosaccharides. Its antigenic activity was determined in the EMA radioimmunoassay.

### Results

*Assay for Epithelial Membrane Antigen*

Establishing a method for the purification of EMA depended on a means of detecting the antigen. Immunodiffusion, immunoelectrophoresis and crossed immunoelectrophoresis of either milk or extracts of the milk fat globule membrane showed that the antigen did not form sharp precipitation lines on reaction with the antibody. Since EMA was discovered from the reaction of an antiserum with sections cut from formalin-fixed paraffin-embedded tissue by an immunohistochemical method (Heyderman et al., 1979), we used this to detect EMA at the start of the purification.

When a crude preparation of EMA had been made, a radioimmunoassay was established. This was used to improve the purification procedure and to prepare EMA which was used in the radioimmunoassay described below.

Skimmed human milk was fractionated with $(NH_4)_2SO_4$ and material of mol. wt $>10^6$ was collected from a column of Sepharose 6B as described below. After extraction with chloroformmethanol (6 parts chloroform: 3 parts methanol: 1 part aqueous solution), the EMA activity was recovered in the aqueous phase. This material was dialysed and used in the assay.

The solution of EMA was diluted in carbonate buffer, pH 9.6, and 50 μl added to small polystyrene wells (Removawell, Dynatech Ltd). Thereafter all dilutions and washings used phosphate buffered saline (0.1 M phosphate, 0.15 M NaCl, 3 mM $NaN_3$, pH 7.5), containing 1% bovine serum albumin. Twenty-five μl of goat anti-EMA serum at a dilution of 1 in 100 was added in separate wells to 100 μl of each test fraction diluted as appropriate. After incubation at ambient temperature overnight, the Removawells were washed thoroughly and 50 μl of the antiserum–test sample mix was added. After 2 h at ambient temperature, the wells were washed, incubated with $^{125}$I-labelled anti-goat γ-globulins (0.2 μCi per well) for 1 h, washed and counted. A particular sample of milk was set aside for use as a standard for this assay. In later work a purified sample of EMA has been used as a standard. Typical curves obtained by this procedure are shown in Figure 1. The data given in the rest of this paper were obtained using this assay.

Any impurity in the EMA preparation to which there was an an antibody in the antiserum would interfere in the assay. To check this, the purer preparations of EMA were used to affinity-purify goat antibodies (see **Materials and methods**) and a radioimmunometric assay was established. Gamma globulins from a rabbit antiserum were absorbed to the Removawells by incubation at pH 9.6. After washing, dilutions of either purified EMA or of the test sample were added to the wells and left overnight. After further washing, the wells were incubated with affinity-purified goat antibodies, washed and finally incubated with $^{125}$I-rabbit anti-goat antibodies. Using this alternative assay, the purification procedure was re-checked. No significant difference was found in the results using the two assays.

**Figure 1** Radioimmunoassay for EMA as described in the text. Bo/B − 1 is plotted against either concentration of pure EMA or dilution of milk on a double log scale where Bo are the counts bound in the absence of EMA and B the counts bound in the presence of the unlabelled antigen.

*Purification procedures*

Table I shows the results obtained during a typical preparation of EMA.

Human milk (1 l), pooled from different donors and after removal of the cream, was brought to 40% saturation with $(NH_4)_2SO_4$ and the precipitate removed by centrifugation. The supernatant (which contained 75% of the EMA activity) was brought to 80% saturation with $(NH_4)_2SO_4$, the precipitate collected, dissolved and dialysed against distilled water, concentrated and made 1% in the detergent Triton X-100. This material was divided into four and each part fractionated on a column of Sepharose 6B, 0.1% Triton X-100, pH 8.0. The active fractions which eluted in a position corresponding to a mol. wt $>10^6$ (Figure 2) were bulked, concentrated and then stirred with 6 vol of chloroform and 3 of methanol. After separation, the organic phase and the precipitated proteins at the interface were discarded . The aqueous phase

**Table I** Stage by stage comparison of amounts of protein[1] and EMA[2] during purification of EMA from 1 l milk

| *Stage of purification* | *Protein (mg)* | *EMA (mg)*[3] |
|---|---|---|
| Skimmed milk | 11,280 | 597 |
| 40–80% $(NH_4)_2SO_4$ precipitate | 7,904 | 340 |
| Sepharose 6B | 710 | 319 |
| Chloroform-methanol extraction | 43 | 266 |
| CM Sephadex | 40.8 | 254 |
| Peanut lectin affinity column | 12.3 | 194 |

[1]Determined using the Bio-Rad assay.

[2]Determined using the radioimmunoassay described in this paper.

[3]This value is calculated assuming that the final purification stage gives pure EMA.

**Figure 2** Chromatography on Sepharose 6B. Material in skimmed milk which was soluble at 40% saturation $(NH_4)_2SO_4$ was made 1% in Triton X-100 and eluted in phosphate buffer, 0.1% Triton X-100, pH 8.0. The arrows show the positions at which different markers start to elute. 1-Dextran Blue (void volume); 2-ferritin (mol. wt = 440 K daltons); 3-bovine serum albumin (mol. wt = 67 K daltons); 4-cytochrome C (mol. wt = 12.5 K daltons). (———) EMA activity as measured in a radioimmunoassay; (– – –) Optical density at 280 nm; (-----) Protein concentration.

was dialysed against water, concentrated, made 0.01 M in acetate, pH 6.0, and applied to a column of the ion exchange resin carboxymethyl Sephadex C-25 equilibrated in the same buffer. The major active fraction was washed off in 160 ml of the starting buffer. The remaining activity and proteins were eluted with 0.25 M NaCl, 0.01 M acetate, pH 6.0 (Figure 3). After extensive dialysis against water, some of this material was freeze dried in order to estimate the dry weight. The remainder was applied to a column of peanut lectin immobilised on Sepharose 4B. Ninety-one percent of the applied activity bound to the lectin and 93% of the bound activity was eluted with 2% galactose. This material was dialysed against water and freeze dried.

Some of the batches of EMA were purified by omitting the ion exchange column and substituting an affinity column of wheat germ lectin bound to Sepharose 4B. EMA activity bound to the column and was eluted with 10% N-acetyl D-glucosamine. After dialysis against distilled water the active material was applied to a column of peanut lectin.

*Chemical and physical properties*

Table II shows the values of the gross composition of four different samples of EMA. The total amount of carbohydrate in the antigen was variable but always constituted the major detected component. Protein composition also varied from one preparation of antigen to another and was typically low. Variation was also found in the value obtained for the protein content of particular EMA preparations depending on the method used for determination. The samples had a high content of inorganic material as demonstrated by the weight of ash found. We have been unable to remove this inorganic material despite extensive dialysis. The phosphate and sulphate contents of one sample of EMA were determined and both were $<1\%$ of total wt. Heating a sample of EMA at 110°C to remove water reduced the wt by only 5%.

The monosaccharide compositions of the 4 preparations of EMA are shown in Table III. Galactose is the major monosaccharide in EMA showing a consistent value of ~40%. This was not an artefact caused by insufficient dialysis after

**Figure 3** Cation exchange chromatography on carboxymethyl-Sephadex. The active material from the column shown in **Figure 2** was applied in 0.01 M acetate, 0.01 M NaCl, pH 6.0 and eluted with this buffer. At the point marked with an arrow the concentration of NaCl was increased to 0.25 M.

affinity purification on peanut lectin since material obtained from the ion exchange column before further purification on the peanut lectin also contained large quantities of galactose as did a sample which was affinity purified on immobilised goat anti-EMA antibodies. N-acetyl-glucosamine is also a major sugar in all of the preparations. Other sugars are present in the antigen in more variable quantities.

When one of the samples of EMA (EMA D1) was treated in sequence with periodate, borohydride and dilute acid (i.e. degraded according to the procedure of Smith (Hay *et al.,* 1965)) a considerable simplification of monosaccharide composition of the antigen was achieved (Table III) with no apparent loss in antigenic activity of the antigen (Figure 4). N-acetyl-glucosamine, galactose and N-acetyl-galactosamine were the only significant remaining sugars and were present in the simple molecular ratio of 2:1:1.

Amino acid analyses of the protein portion showed considerable variation. A consistent feature was the absence of tyrosine which accords with the difficulty encountered in attempting to label the antigen with $^{125}I$.

The process of purification reduced the mol. wt of EMA, although it still had a very heterogeneous spread of mol. wts between $10^6$ and $5.10^4$

**Table II** Composition (by wt) of purified EMA preparations

| *EMA preparation* | *EMA M4* | *EMA D1* | *EMA S18* | *EMA S21* |
|---|---|---|---|---|
| Carbohydrate (%) | 58.0 | 22.9 | 24.5 | 40.0 |
| Protein (%) | 14.0[1]; 16.0[2] | 13.6[1]; 6.6[2] | 6.6[2] | 4.7[2] |
| Ash (%) | 9.7 | ND | 18.6 | 16.8 |

[1]Determined using the Lowry protein assay.
[2]Determined from the amino-acid analysis of the sample.
ND = not determined.

**Table III** Monosaccharide analysis (mole %) of EMA and Smith-degraded EMA D1

| *EMA preparation* | *EMA M4* | *EMA D1* | *EMA S18* | *EMA S21* | *Smith degraded EMA D1* |
|---|---|---|---|---|---|
| Fucose | 3.7 | 4.7 | 4.5 | 7.1 | 1.0 |
| Mannose | 0.9 | 3.8 | 15.2 | 5.9 | 1.8 |
| Glucose | 2.7 | 13.6 | 12.8 | 6.2 | 2.6 |
| Galactose | 42.9 | 42.8 | 38.1 | 42.6 | 24.5 |
| GlcNAc | 32.3 | 19.9 | 18.6 | 26.8 | 48.9 |
| GalNAc | 16.8 | 12.7 | 11.0 | 11.5 | 21.1 |
| Sialic acid[1] | 1.7 | 2.5 | ND | ND | none |

[1]Determination using the colorimetric method of Warren (1959).
ND = not determined.

**Figure 4** Radioimmunoassay of EMA (x) and Smith-degraded EMA (●). Bo/B−1 is plotted against the concentration of either EMA or modified EMA on a double log scale where Bo are the counts bound in the absence of antigen and B the counts bound in the presence of antigen.

**Figure 5** Purified EMA on a column of Sepharose-CL 4B. Eluted in phosphate buffer, 0.1% Triton X-100. The arrows show the positions at which different markers start to elute. From left to right, blue dextran (void volume); IgM (mol. wt = 800 K daltons); IgG (mol. wt = 150 K daltons); bovine serum albumin (mol. wt = 67 K daltons).

(Figure 5). The activity of EMA was unaffected by placing a solution in a boiling water bath for 2 min. It was also unaffected by heating to 90°C for 2 min in the presence of 3% SDS and 1% mercaptoethanol. On a polyacrylamide gel at pH 8.6 the EMA activity had a low electrophoretic mobility in the presence or absence of SDS. Figure 6 shows a comparison of EMA activities in skimmed milk and a preparation of EMA. The latter had a wide range of mobilities consistent with a larger spread of mol. wt.

Treatment with trypsin (1 mg per 10 mg protein) for 17 h at 37°C had no effect on the EMA activity. Treatment with pronase (1 mg per 10 mg protein) for 2 h at 37°C also had no effect but if the incubation was extended to 17 h, there was a lowering of activity. This is an excessive length of treatment for this enzyme and the effect could have been due to trace impurities in the pronase sample and not its proteolytic activity. EMA could be exposed for up to 4 h to either 1 M acetic acid or 6 M guanidine without effect but exposure for 18 h or more did lead to loss of activity. If exposure to either acetic acid or guanidine is not excessive, then columns in either of these reagents can give a considerable purification of EMA at an early stage in the procedure. However, these columns removed little that was not removed by extraction with chloroform/methanol.

As well as binding to peanut lectin, EMA also bound to wheat germ lectin. Despite its mannose

**Figure 6** 6½% polyacrylamide gels run in SDS. The direction of migration is from left to right. The small peak of activity at the end of the gel marks the solvent front and is due to the higher concentration of SDS at this position interfering in the radioimmunoassay. (a) Skimmed milk; (b) Purified EMA.

content, EMA did not react with Concanavalin A although the active material which precipitated with 40% $(NH_4)_2SO_4$ did bind to Con A; this Con A reactivity could be removed by treatment with trypsin.

### Discussion

Antibodies to EMA have now been proved to be of value in diagnostic tumour histopathology and have demonstrated the presence of the antigen on different epithelial tissues. However, the nature of the molecules which carry the antigenic determinants and the structures of the determinants are not known. It would be of interest to know if the molecules which carry the determinants are the same in each tissue and also whether there are any variations in the determinants carried by different tissues and whether their pattern changes in neoplasia.

The discovery of EMA in a soluble form in human milk presented an opportunity to purify the antigen and thereby to examine the structure of the molecules. As our attempts at purification proceeded, it became clear that the antigenic determinants were not carried on one well-defined molecule but that we were dealing with a heterogeneous set. This is evident from the spread of apparent mol. wt on the Sepharose column. EMA is present in the membrane of the milk fat globules which have arisen from the apical membranes of the secretory cells of the breast and the soluble antigen is probably formed by degradation of milk fat globule membranes in the milk. This degradative process might explain the variability in the protein content. The variation from one preparation to another was far higher than would be expected even taking into consideration the low protein content of the final product. Batches of milk from different women might vary appreciably in the extent to which the antigen had been degraded. An alternative explanation for the variability in protein is that much of this material is impure. This seems unlikely since on SDS-polyacrylamide gels the only protein band observed (and that was very weak) co-eluted with the EMA activity as measured by the radioimmunoassay. We have found previously that such gels would separate EMA from casein with which the antigen had been co-purified (Ormerod *et al.*, 1982).

During the process of isolation there was further breakdown of the antigen as is shown by a comparison of Figures 2 and 5. There was little evidence that this was due to proteolysis and it may have been caused by breaking strong hydrophobic links.

The most effective purification steps were the chloroform-methanol extraction and the peanut lectin affinity column. The precipitation with 40% $(NH_4)_2SO_4$ did not increase the EMA/protein ratio in itself but removed a lot of material of high mol. wt which would have interfered with the purification on the Sepharose 6B column. The ion-exchange column did not improve the ratio of EMA to protein but it did remove some non-proteinaceous material and was therefore retained in the purification procedure.

Two other perplexing features of the purification were our inability to account for all of the material present and the high ash content. With different samples we could account for between 45 and 80% of the total wt in terms of carbohydrate, protein and inorganic material. It is unlikely that much lipid is present as a lipid would be expected to partition into the organic phase during the chloroform-methanol extraction. The ash present was not phosphate or sulphate. Whatever its composition, it was firmly bound to the sample since the EMA had been affinity purified on peanut lectin after it had last been dissolved in a buffer. Thereafter it was handled in distilled water.

While the protein content of the preparations of EMA was low, carbohydrate constituted the major identified part of the preparations with galactose and N-acetyl-glucosamine being the predominant sugars; five other sugars, however, were also identified. Smith degradation considerably simplified the carbohydrate composition leaving N-acetyl-glucosamine, galactose and N-acetyl-galactosamine, in the molecular ratio of 2:1:1, respectively; the modified antigen retained its antigenic activity. This may mean that the major antigenic determinants contain a combination of these sugars particularly as removal of the majority of the sugars from the antigen using neuraminidase and a mixture of glycosidases isolated from *Trichomonas foetus* (Watkins, 1966) caused a large reduction in antigenic activity (unpublished results) whereas treatment of EMA with trypsin caused little loss in EMA activity. Although the Smith degradation of EMA would be expected to destroy carbohydrate determinants, it may also expose cryptic carbohydrate structures similar to the ones which originally bound to the antiserum.

Recently, Shimizu & Yamuchi (1982) have extracted a mucin-like glycoprotein from the human milk fat globule membrane using a mixture of deoxycholate, urea and mercaptoethanol. This glycoprotein has many properties similar to EMA but it will only be possible to confirm the identity of the two materials by comparing them in a radioimmunoassay.

It is clear from our results that our polyclonal antisera, while identifying a ubiquitous structure associated with epithelial differentiation and of practical importance in cancer research, are not reacting with a simple molecular species. We have probably isolated a set of products resulting from the breakdown of a much larger component of the membrane. We present the purification method developed now because it produces a product which can be used to absorb antisera (an important control in immunohistochemical work), it forms the basis of our radioimmunoassay which can be used to detect EMA released in the plasma of patients with breast cancer (Ormerod *et al.*, in preparation), and it gives the starting material for more detailed work on the structure of the antigenic determinants. This will be achieved by a variety of methods including selective degradation of the carbohydrate structure. There are now, in addition to the polyclonal antisera used for the work described in this paper, 5 monoclonal antibodies available, one raised in the Ludwig Institute for Cancer Research (Foster *et al.*, 1982), 2 in the Imperial Cancer Research Fund (Taylor–Papadimitriou *et al.*, 1981) and 2 in the Institute of Cancer Research (Summerhayes & Pocock, work in progress). These antibodies are currently being used to explore the different epitopes of the antigen.

We thank Prof. A.M. Neville for his helpful encouragement and advice during the work and Dr. Anne Neville for organising the supply of, and the nursing mothers for supplying, the human milk. We also thank the following people: Dr. E. Heyderman for her immunohistochemical testing for EMA in the early stages of the work, Mr. C.L. Day and Dr. A.M. Bukhari for their skilled technical assistance, and Dr. M. Warburton for the amino acid analyses. The work was supported by grants from the Medical Research Council. One of us (M.N.M.) thanks the Consejo Nacional de Investigaciones Cientificas y Technicas de la Republica Argentina for her support.

### References

BAMFORD, P.N., ORMEROD, M.G., SLOANE, J.P. & WARBURTON, M.J. (1983). An immunohistochemical study of the distribution of epithelial antigens in the uterine cervix. *Obstet. Gynecol.*, **41**, 603.

CERIANI, R.L., THOMPSON, K., PETERSON, J.A. & ABRAHAM, S. (1977). Surface differentiation antigens of human mammary epithelial cells carried on the human milk fat globule. *Proc. Natl Acad. Sci.*, **74**, 582.

CLAMP, J.R., BHATTI, T. & CHAMBERS, R.E. (1971) The determination of carbohydrate in biological materials by gas liquid chromatography. *Meth. Biochem. Anal.*, **19**, 229.

DEARNALEY, D.P., ORMEROD, M.G., SLOANE, J.P. & 5 others (1983). Detection of isolated mammary carcinoma cells in the marrow of patients with primary breast cancer, *Proc. R. Soc. Med.*, **76**, 359.

DEARNALEY, D.P., SLOANE, J.P., ORMEROD, M.G., STEELE, K & 6 others (1981). Increased detection of mammary carcinoma cells in marrow smears using antisera to epithelial membrane antigen. *Br. J. Cancer*, **44**, 85.

FOSTER, C.S., EDWARDS, P.A.W., DINSDALE, E.A. & NEVILLE, A.M. (1982). Monoclonal antibodies to the human mammary gland. I. *Virchows Arch.* (*Pathol. Anat.*) **394**, 279.

HAY, G.W., LEWIS, B.A. & SMITH, F. (1965). Periodate oxidation of polysaccharides: General procedures. In: *Methods in Carbohydrate Chemistry, Vol. 5,* (Ed. Whistler) p. 357. Academic Press: New York.

HEYDERMAN, E., STEELE, K. & ORMEROD, M.G (1979). A new antigen on the epithelial membrane: its immunoperoxidase localisation in normal and neoplastic tissues. *J. Clin. Pathol.*, **32**, 35.

LOWRY, O.H., ROSENBROUGH, N.J., FARR, A.L. & RANDALL, R.J. (1951). Protein measurement with the Folin phenol reagent. *J. Biol. Chem.*, **193**, 265.

ORMEROD, M.G., BUSSULATI, G., SLOAN, J.P., STEELE, K. & GUGLIOTTA, P. (1982). *Virchows. Arch.*, **397**, 327.

ORMEROD, M.G., MONAGHAN, P., EASTY, D. & EASTY, G.C. (1981). Asymmetrical distribution of epithelial membrane antigen on the plasma membrane of human breast cell lines in culture. *Diagn. Histopathol.*, **4**, 89.

SHIMIZU, M. & YAMAUCHI, K. (1982). Isolation and characterisation of mucin-like glycoprotein in human milk fat globule membrane. *J. Biochem.*, **91**, 515.

SLOANE, J.P., HUGHES, F. & ORMEROD, M.G. (1983). An assessment of the value of epithelial membrane antigen and other epithelial markers in solving diagnostic problems in tumour histopathology. *Histochem. J.*, **15**, 645.

SLOANE, J.P., ORMEROD, M.G., CARTER, R.L., GUSTERSON, B.A. & FOSTER, C.S. (1982). An immunocytochemical study of the distribution of epithelial membrane antigen in normal and disordered squamous epithelium. *Diagn. Histopathol.*, **5**, 11.

SLOANE, J.P. & ORMEROD, M.G. (1981). Distribution of epithelial membrane antigen in normal and neoplastic tissues and its value in diagnostic tumor pathology. *Cancer*, **47**, 1786.

SLOANE, J.P., ORMEROD, M.G., IMRIE, S.F. & COOMBES, R.C. (1980). The use of antisera to epithelial membrane antigen in detecting micrometastases in histological sections. *Br. J. Cancer*, **42**, 392.

TAYLOR-PAPADIMITRIOU, J., PETERSON, J.A., ARKLIE, J., BURCHELL, J., CERIANI, R.L. & BODMER, W.F. (1981). Monoclonal antibodies to epithelium-specific components of the human milk fat globule membrane: production and reaction with cells in culture. *Int. J. Cancer*, **28**, 17.

TO, A., COLEMAN, D.V., DEARNALEY, D.P., ORMEROD, M.G., STEELE, K., & NEVILLE, A.M. (1981). Use of antisera to epithelial membrane antigen for the cytodiagnosis of malignancy in serous effusions. *J. Clin. Pathol.*, **34**, 1326.

TO, A., DEARNALEY, D.P., ORMEROD, M.G., CANTI, G. & COLEMAN, D.V. (1982). Epithelial Membrane Antigen: Its use in the diagnosis of malignancy in serous effusions. *Am. J. Clin. Pathol.*, **78**, 214.

VON KLEIST, S., CHAVANEL, G. & BURTIN, P. (1972). Identification of an antigen from normal human tissue that cross-reacts with carcinoembryonic antigen. *Proc. Natl Acad. Sci.*, **69**, 2492.

WATKINS, W.M. (1966). Enzymes that destroy blood-group specificity. *Methods Enzymol.*, **8**, 700.

WESTWOOD, J.H. & THOMAS, P. (1975). Studies on the structure and immunological activity of carcinoembryonic antigen—the role of disulphide bonds. *Br. J. Cancer*, **32**, 708.